# Combining OpenStreetMap with Satellite Imagery to Enhance Cross-View Geo-Localization

**DOI:** 10.3390/s25010044

**Published:** 2024-12-25

**Authors:** Yuekun Hu, Yingfan Liu, Bin Hui

**Affiliations:** 1 Key Laboratory of Opto-Electronic Information Processing, Chinese Academy of Sciences, Shenyang 110016, China; huyuekun@sia.cn (Y.H.); liuyingfan@sia.cn (Y.L.); 2 Shenyang Institute of Automation, Chinese Academy of Sciences, Shenyang 110016, China; 3 University of Chinese Academy of Sciences, Beijing 100049, China

**Keywords:** cross-view geo-localization, OpenStreetMap, satellite imagery, data fusion

## Abstract

Cross-view geo-localization (CVGL) aims to determine the capture location of street-view images by matching them with corresponding 2D maps, such as satellite imagery. While recent bird’s eye view (BEV)-based methods have advanced this task by addressing viewpoint and appearance differences, the existing approaches typically rely solely on either OpenStreetMap (OSM) data or satellite imagery, limiting localization robustness due to single-modality constraints. This paper presents a novel CVGL method that fuses OSM data with satellite imagery, leveraging their complementary strengths to enhance localization robustness. We integrate the semantic richness and structural information from OSM with the high-resolution visual details of satellite imagery, creating a unified 2D geospatial representation. Additionally, we employ a transformer-based BEV perception module that utilizes attention mechanisms to construct fine-grained BEV features from street-view images for matching with fused map features. Compared to state-of-the-art methods that utilize only OSM data, our approach achieves substantial improvements, with 12.05% and 12.06% recall enhancements on the KITTI benchmark for lateral and longitudinal localization within a 1-m error, respectively.

## 1. Introduction

Vision-based localization [1,2,3,4,5]—the process of determining a camera’s pose using visual data—is a fundamental task in computer vision with extensive applications in robotics, augmented reality, and autonomous vehicles. The selection of map data plays a pivotal role in developing robust and accurate localization techniques. Traditionally, detailed 3D maps generated from photogrammetric methods [1] have served as the primary source of geospatial information for precise localization. However, creating high-quality 3D maps presents significant challenges: it requires specialized equipment, demands substantial computational resources for data processing and map construction, and produces large datasets that pose difficulties in storage, transmission, and real-time access, particularly in resource-constrained environments.

**Figure 1:**
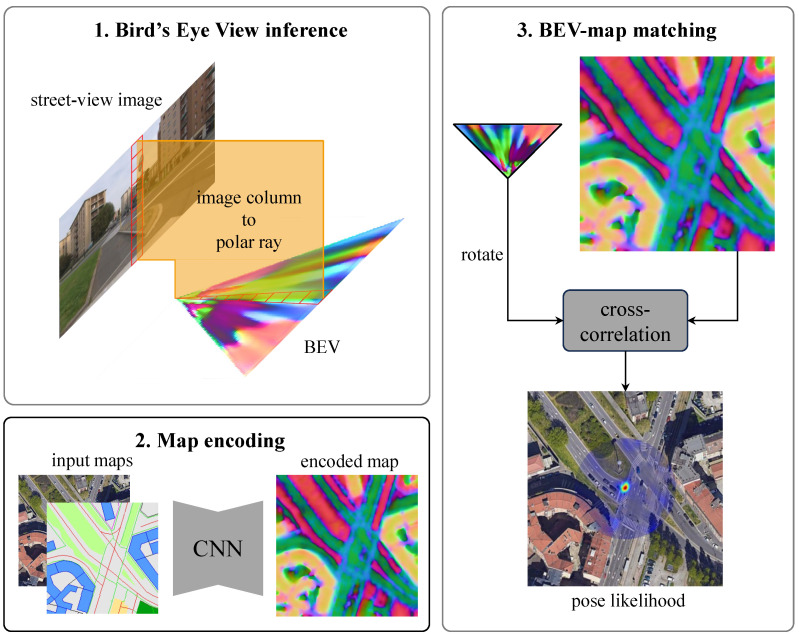
Overview of bird’s eye view (BEV)-based cross-view geo-localization (CVGL) method. Street-view image is used to form a BEV, while input maps are encoded for BEV-map matching to obtain a pose likelihood. Satellite maps data: Google, ©2024 Airbus, CNES/Airbus, Maxar Technologies [11].

To address these challenges, researchers have increasingly turned to 2D-map-based approaches, particularly cross-view geo-localization (CVGL). CVGL associates street-view images with corresponding top-down maps to determine the capture location. To handle the substantial viewpoint and appearance disparities between the two perspectives, recent advances in this field have achieved meter-level accuracy by constructing a bird’s eye view (BEV) map from street-view images [6,7]. As shown in Figure 1, the BEV refers to a top-down view that is consistent with 2D maps and is matched with a map to predict the relative pose with three degrees of freedom (3-DoF).

In contrast, the impact of map data on localization performance remains understudied [8]. Two predominant types of 2D maps with precise geo-referencing capabilities are satellite imagery and OpenStreetMap (OSM) [9]. Satellite imagery, leveraging advances in remote sensing technology, provides high-resolution, temporally current visual data of the Earth’s surface at increasingly cost-effective rates. This reduces the dependence on extensive ground-based mapping campaigns while maintaining high data quality. In contrast, OSM offers a complementary approach by providing abstracted representations that focus on the structural and semantic aspects of the environment. As a globally available, open-source platform, OSM encodes the 2D location, geometry, and categorical information of map elements while abstracting away appearance variations. This semantic richness, combined with its worldwide accessibility, has established OSM as a valuable resource for localization applications.

**Figure 2:**
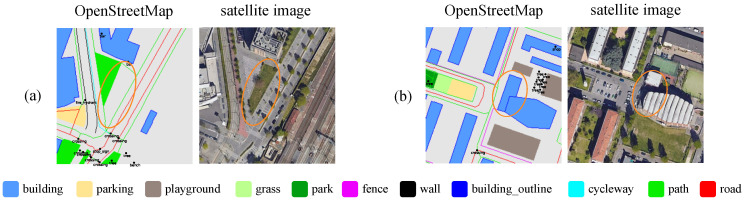
(**a**) shows that OpenStreetMap (OSM) lacks some semantics like grass, while (**b**) illustrates how shadows can make building identification difficult in satellite imagery. Satellite maps data: Google, ©2024 Airbus, CNES/Airbus, Maxar Technologies [11].

Despite their individual advantages, both OSM data and satellite imagery present challenges for CVGL. As shown in Figure 2 (OSM visualizations follow the color scheme of OrienterNet [6]), OSM’s reliance on manual data entry introduces variability in accuracy and completeness, which can vary significantly between regions depending on contributor engagement [10]. Satellite imagery, while providing rich visual information, may suffer from temporal discrepancies due to changes in land cover, lighting conditions (shown in Figure 2) or seasonal variations, thus affecting the robustness of CVGL. Moreover, the detailed visual information sometimes does not correspond to street-level observations. For instance, satellite imagery reveals the rooftops of buildings, which are not visible in street-view images, thereby challenging the robustness of matching.

Recognizing the complementary strengths and limitations of OSM data and satellite imagery, we propose integrating these two modalities for more robust CVGL. By fusing the semantic richness and structural information of OSM with the visual fidelity of satellite imagery, we aim to create a more robust and versatile 2D geospatial representation that enhances localization performance. Specifically, we present a method that encodes and fuses OSM data and satellite imagery to form a unified map representation within the OrienterNet framework [6]. As illustrated in Figure 1, the OrienterNet is an advanced visual localization method that utilizes OSM data and BEV semantic matching for CVGL. We extend it by integrating satellite imagery into its localization framework, enabling the system to leverage both semantic and visual information derived from remote sensing data. The proposed method encodes multiscale feature maps and fuses them based on a spatial attention mechanism [12], which dynamically modulates the contribution of each data source.

Furthermore, to obtain fine-grained BEV features from street-view images, we replace the convolutional neural network (CNN)-based BEV inference with a transformer-based model. While CNNs typically predict depth distributions and assign image features to corresponding BEV positions based on this depth information, our transformer-based approach uses position queries to implicitly learn a proxy for depth. This allows for iterative aggregation of information from image columns through cross-attention and refinement self-attention mechanisms [13], resulting in more refined and detailed BEV features.

In summary, our contributions are as follows:

- We propose a novel fusion method that seamlessly integrates OpenStreetMap data with high-resolution satellite imagery to create a unified map representation. Our approach effectively encodes and combines multi-scale feature maps from both data sources, enhancing semantic richness and visual fidelity.
- We introduce a transformer-based BEV perception module that constructs BEV features by aggregating information from image columns. Unlike CNN-based methods that learn depth distributions, our transformer-based model utilizes position queries and attention mechanisms to implicitly and efficiently learn a proxy for depth, resulting in iteratively refined and fine-grained BEV features.
- Our results demonstrate that the fused map features significantly enhanced localization accuracy compared to using either OSM data or satellite imagery alone, substantially outperforming the previous works on localization accuracy in driving scenarios. Our analysis, supported by visualizations, reveals improvements in scenarios where one data source is restricted, for example, in areas where OSM data are incomplete or outdated or where buildings are difficult to identify in satellite images.

The remainder of this paper is organized as follows. The related work is presented in Section 2. In Section 3, we detail the proposed method. Section 4 provides the experimental setup and results. The discussion and conclusion are covered in Section 5 and Section 6, respectively.

## 2. Related Work

CVGL has been extensively studied within the computer vision community, with numerous approaches developed to estimate a camera’s position and orientation using visual data. In this section, we review the related work in three key areas: image-retrieval-based CVGL, cross-view camera pose estimation, and the utilization of map data for localization.

### 2.1. Image-Retrieval-Based Cross-View Geo-Localization

Traditional cross-view geo-localization methods [14] treat the problem as an image retrieval task, aiming to identify the most similar aerial image corresponding to a given ground-level query image. Early approaches employed Siamese-like networks with separate branches for ground and aerial images to address the significant domain discrepancies [4,15]. These networks encode images into global descriptors, enabling similarity measurements despite drastic viewpoint differences.

To bridge the modality gap between ground and aerial images, several strategies have been proposed. Regmi and Shah [16] utilized generative adversarial networks (GANs) [17] to synthesize aerial images from ground-level views, thereby reducing domain discrepancies by providing more aligned representations. Similarly, Shi et al. [15] proposed spatial-aware feature aggregation (SAFA), which applied polar transformations to aerial images to better align them with ground-level perspectives.

In general, image-retrieval-based methods often serve as substitutes for noisy GPS in localization tasks but do not provide orientation information. To achieve accurate localization, these methods heavily depend on the sampling density of the map image database [18]. Recently, coarse location priors derived from GPS or initial image retrieval have enabled cross-view pose estimation to achieve meter-level accuracy.

### 2.2. Cross-View Camera Pose Estimation

Cross-view camera pose estimation methods seek to determine the exact location and orientation of a ground-level image relative to an overhead map. Shi et al. [18] first proposed a method that diverges from traditional image retrieval by projecting overhead map features into the ground view using differentiable homography transformations. By minimizing the differences between spatially aligned map features and ground-level image features through optimization techniques, they accurately determined the relative pose between the camera and the map.

Sarlin et al. [6] introduced OrienterNet, which leverages BEV perception [19,20] and OpenStreetMap data for pose estimation using a CNN. OrienterNet infers a BEV representation from a street-view image and matches it with a neural map derived from OSM data, achieving state-of-the-art localization results across various scenarios. This framework mimics human spatial reasoning; however, inferring BEV representations from monocular images presents significant challenges due to the inherent ambiguity of projecting 3D information onto a 2D plane. Accurate BEV inference often requires additional information, such as LiDAR data [21] or sophisticated models like Transformers [13,20] to resolve depth ambiguities. In contrast, recent advancements have made less improvement in 2D maps for CVGL [8].

### 2.3. Map Data Used for Localization

Map data serves as a critical reference for visual localization tasks, with various types of maps being utilized, including 3D maps constructed from ground images, satellite imagery, and OpenStreetMap data.

High-precision localization frequently relies on 3D maps created using structure-from-motion (SfM) techniques [1]. These maps consist of sparse point clouds generated by matching features across multiple viewpoints. Algorithms based on feature matching can estimate centimeter-accurate 6-DoF poses by finding correspondences between the query image and the 3D maps [22,23]. While highly accurate, creating and maintaining such maps is resource-intensive, resulting in large memory footprints.

Satellite imagery offers a global perspective and has been widely utilized in CVGL research [18]. A significant body of work focuses on cross-view ground-to-satellite localization, primarily operating within the vision modality. Consequently, proposed methods often concentrate on overcoming the view gap inherent between ground and satellite images.

OpenStreetMap provides freely available, globally accessible map data rich in semantic information about roads, buildings, and other geographic features. Unlike traditional image-based maps, OSM data discard appearance details, retaining only the 2D locations, shapes, and types of map elements. Earlier works typically used a native RGB map tiles [24,25], which is straightforward for human reading but inefficient in capturing semantic information. Recently, Sarlin et al. [6] enhanced this by introducing a rasterization for vector-based OSM data, effectively leveraging the comprehensive semantic classes available in OSM.

Our method extends OrienterNet by incorporating a multi-modality map encoding that integrates detailed visual information from satellite imagery with high-level semantic data from OSM. This fusion creates a more comprehensive and robust map reference, thereby enhancing the performance of CVGL.

## 3. Methods

Our approach is depicted in Figure 1. The framework comprises three sequential components designed to achieve precise localization:

- Transformer-based BEV Perception Module: Transforms monocular street-view images into BEV representation $B\in R^{X\times D\times N}$, distributed across an X×D grid with *N*-dimensional features with grid size of Δ×Δ (Section 3.1).
- Map Fusion Module: Encodes and fuses OpenStreetMap data with satellite imagery to form unified map features M (Section 3.2).
- Pose Estimation Module: Combines BEV and map features to estimate a 3-DoF pose ξ=(x,y,θ), where $\left(x,y\right)\in R^{2}$ represents location and θ∈(−π,π] denotes heading angle (Section 3.3).

### 3.1. BEV Inference

The BEV perception begins with a street-view image captured by a calibrated pinhole camera. The image undergoes rectification to eliminate roll and pitch angles, ensuring horizontal alignment of the optical axis. This configuration creates a crucial geometric relationship: BEV pixels aligned along the same direction form polar rays, with each ray corresponding to a vertical column in the image [6,26].

To effectively map spatial information from image columns to polar rays, the architecture employs a transformer decoder. A CNN extracts image features $F\in R^{U\times V\times N}$, where *U* and *V* represent spatial dimensions, and *N* denotes the feature dimension. For each image feature column *u*, source sequence $S_{u}\in R^{V\times N}$ is extracted and added with sinusoidal positional encoding [13] to preserve spatial context.

**Figure 3:**
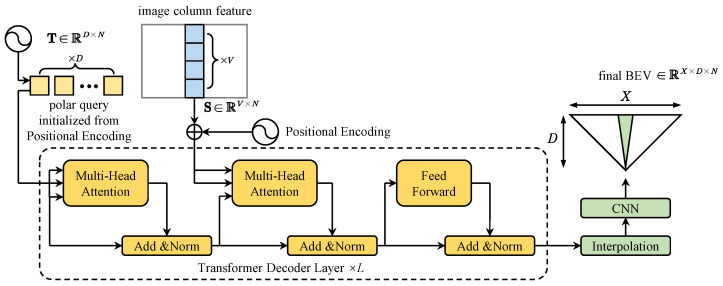
Transformer decoder architecture for BEV inference, illustrating the mapping from image columns to BEV polar rays using self-attention and cross-attention mechanisms.

As shown in Figure 3, the polar query $T_{u}\in R^{D\times N}$ is used to aggregate information from the corresponding $S_{u}$ to form a polar ray, where *D* represents the discretized depth range with interval Δ, e.g., 1 m per pixel. Each polar query is first initialized from sinusoidal positional encoding corresponding to *D* depth positions, allowing the model to effectively infer spatial positions along these rays.

The mapping from image features to BEV polar rays leverages a multi-head attention (MHA) mechanism [13] within the transformer decoder. Specifically:

- Self-Attention: Applied to the target polar rays T to refine the BEV representation.
- Cross-Attention: Integrates features from the source image column S into T.
- Feed-Forward Network (FFN): Further processes the integrated information.

These operations are iteratively applied over *L* layers as follows:

(1) $$\begin{array}{cc} {\mathrm{SA}}_{u}^{\left(l\right)} & =\mathrm{LN}\left(\mathrm{MHA}\left(T_{u}^{\left(l-1\right)},T_{u}^{\left(l-1\right)},T_{u}^{\left(l-1\right)}\right)+T_{u}^{\left(l-1\right)}\right) \end{array}$$

(2) $$\begin{array}{cc} {\mathrm{CA}}_{u}^{\left(l\right)} & =\mathrm{LN}\left(\mathrm{MHA}\left({\mathrm{SA}}_{u}^{\left(l\right)},S_{u},S_{u}\right)+{\mathrm{SA}}_{u}^{\left(l\right)}\right) \end{array}$$

(3) $$\begin{array}{cc} T_{u}^{\left(l\right)} & =\mathrm{LN}\left(\mathrm{FFN}\left({\mathrm{CA}}_{u}^{\left(l\right)}\right)+{\mathrm{CA}}_{u}^{\left(l\right)}\right) \end{array}$$

where LN(·) indicates the layer normalization operation [27]; the MHA is computed as:

(4) $$\left[{\mathrm{head}}_{1};{\mathrm{head}}_{2};\ldots ;{\mathrm{head}}_{h}\right]W^{O}$$

where each attention head employs the scaled dot-product attention mechanism [13]:

(5) $${\mathrm{head}}_{i}=\mathrm{Attention}\left(QW_{i}^{Q},KW_{i}^{K},VW_{i}^{V}\right)$$

with projection matrices $W_{i}^{Q}\in R^{N\times d_{Q}}$, $W_{i}^{K}\in R^{N\times d_{K}}$, $W_{i}^{V}\in R^{N\times d_{V}}$, and $W^{O}\in R^{N\times N}$. For self-attention, $Q=K=V=T_{u}$. In the case of cross-attention, K and V are replaced with $S_{u}$. Here, $d_{Q}=d_{K}=d_{V}=\frac{N}{h}$, ensuring dimensional consistency across attention heads. After *L* layers, the final BEV representation is obtained as $T_{u}^{\left(L\right)}$. The polar BEV representation $B_{p}\in R^{U\times D\times N}$ is formed by stacking all polar rays:

(6) $$B_{p}=\left[T_{1}^{\left(L\right)};T_{2}^{\left(L\right)};\ldots ;T_{U}^{\left(L\right)}\right]$$

This sequence-to-sequence process maintains geometric imaging priors along the azimuth axis by constructing the polar BEV. Subsequently, a linear interpolation is performed along the azimuth axis to convert the polar BEV $B_{p}$ to a Cartesian grid BEV $B\in R^{X\times D\times N}$, where *X* is the number of columns spaced by interval Δ. A small CNN then refines the final BEV representation.

### 3.2. Map Fusion

To effectively integrate OpenStreetMap data with satellite imagery, we introduce a dual-stream network to encode multi-scale feature maps, followed by a fusion module to fuse each scale, which are decoded into unified map features with original resolution.

**Figure 4:**
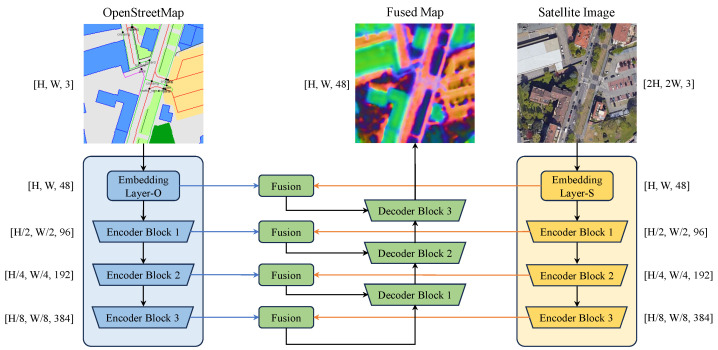
The map encoding pipeline adopts a dual-stream architecture based on U-Net [28] to fuse OSM and satellite imagery. Shapes within square brackets represent tensor dimensions, specifically height, width, and channels.

As illustrated in Figure 4, the encoder comprises two parallel processing streams, each beginning with an embedding layer that encodes a 48-channel feature map.The OSM input includes elements categorized as areas, lines, or points [6]. These elements are first rasterized at a fixed resolution. The rasterized areas, lines, and points are assigned to three separate channels. In each channel, the blank area is represented by a value of zero, while different semantic classes are indicated by non-zero integers. The OSM embedding layer (Embedding Layer-O) maps each set of integer indices corresponding to a channel into 16-dimensional vectors. By embedding the three channels separately, three distinct 16-channel feature maps are obtained. These feature maps are then concatenated to form a 48-channel feature map, which serves as the output of the OSM embedding layer.

Meanwhile, the satellite image is treated as an RGB input with twice the resolution of the rasterized OSM data. This image is processed by the satellite embedding layer (Embedding Layer-S) using a 7×7 convolutional layer with a stride of 2. This process produces a 48-channel feature map that maintains the same spatial resolution as the OSM feature map.

These embedding layers are followed by three sequential encoder blocks that extract hierarchical features at multiple scales. Information from both modalities is integrated through fusion modules: one after the initial embedding layer and additional modules following each encoder block. The fused features are subsequently passed through three decoder blocks, which progressively restore spatial resolution while maintaining the integrated information from both streams. This symmetric encoder–decoder structure [28] ensures balanced feature extraction and refinement, producing rich map features that capture both the geometric precision of satellite imagery and the semantic structure of OSM data.

**Figure 5:**
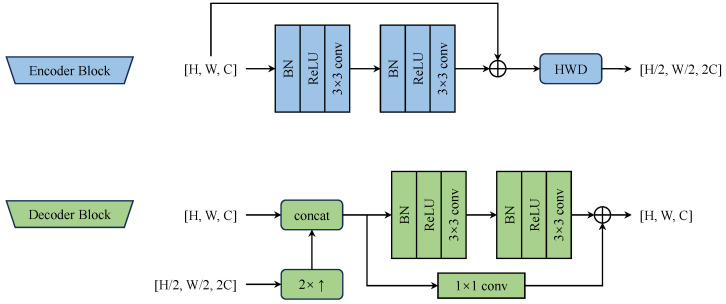
Encoder and decoder block architecture utilizing residual blocks and Haar wavelet downsampling (HWD).

As depicted in Figure 5, each encoder block processes an input feature map with *C* channels through residual blocks, followed by a Haar wavelet-based downsampling (HWD) module [29]. The HWD employs a Haar wavelet transform to reduce spatial resolution while preserving feature information. Unlike traditional downsampling methods that may discard spatial details, the Haar wavelet decomposition captures both coarse and fine-grained features through four complementary components:

- LL: Low-frequency approximation preserves dominant structural information ($\frac{H}{2}\times \frac{W}{2}$).
- LH: Horizontal detail captures edge features along rows ($\frac{H}{2}\times \frac{W}{2}$).
- HL: Vertical detail preserves edge features along columns ($\frac{H}{2}\times \frac{W}{2}$).
- HH: Diagonal detail maintains corner and texture information ($\frac{H}{2}\times \frac{W}{2}$).

These wavelet components are concatenated and processed through a 1×1 convolutional layer to map the 4C channels to 2C channels:

(7) $$Y={\mathrm{Conv}}_{1\times 1}\left(\mathrm{ReLU}\left(\mathrm{BN}\left([\mathrm{LL},\mathrm{LH},\mathrm{HL},\mathrm{HH}]\right)\right)\right)$$

where BN denotes batch normalization [30].

Our fusion module employs a spatial attention mechanism adapted from [12] to dynamically integrate features from both modalities:

(8) $$F=M_{O}⊙A_{\mathrm{att}}+M_{S}⊙\left(1-A_{\mathrm{att}}\right)$$

where ⊙ denotes element-wise multiplication, F represents the fused features, $M_{O}$ and $M_{S}$ denote the OSM and satellite features, respectively, and $A_{\mathrm{att}}$ is a learned spatial attention map. The attention weights are computed as:

(9) $$A_{\mathrm{att}}=\sigma \left({\mathrm{Conv}}_{1\times 1}\left(\mathrm{ReLU}\left(\mathrm{BN}\left([M_{O},M_{S}]\right)\right)\right)\right)$$

where σ denotes the sigmoid activation function. This attention mechanism adaptively emphasizes the most informative aspects of each modality at different spatial locations.

The decoder is also shown in Figure 5, where 2×↑ denotes bilinear upsampling. The decoder mirrors the encoder structure with three decoder blocks, progressively upsamples the fused features and then integrates multi-scale map features, progressively yielding the final map features.

### 3.3. Pose Estimation and Loss Function

BEV-based localization methods typically estimate camera pose likelihood through BEV-to-map feature matching [6,7]. Given a discrete set of candidate poses X, typically constrained by noisy GPS measurements, each pose is represented as ξ=(x,y,θ). BEV features B are first upsampled to match the resolution of map features, and both are linearly projected to *c* channels to reduce dimensionality. For a given pose ξ, BEV features are transformed into map coordinates via the rigid transformation $T_{\xi }$, parameterized by ξ, resulting in $T_{\xi }\left(B\right)$.

The cross-correlation score of a pose ξ is computed as the inner product between the transformed BEV features and the map features M:

(10) $$C\left[\xi \right]=\frac{1}{\left|\Omega \right|}\langle T_{\xi }\left(B\right),M\rangle$$

where |Ω| denotes the number of valid pixels in $T_{\xi }\left(B\right)$. Consequently, the cross-correlation scores are normalized to obtain the pose likelihood:

(11) $$P\left[\xi \right]=\frac{\exp C[\xi ]}{\sum_{\xi^{\prime }\in X}\exp C\left[\xi^{\prime }\right]}$$

Finally, the optimal pose $\xi^{*}$ is estimated by maximizing the likelihood:

(12) $$\xi^{*}=\underset{\xi \in X}{\mathrm{argmax}}P\left[\xi \right]$$

Notably, the cross-correlation between a rotated BEV and the map is treated as a 2D convolution. This operation is efficiently computed in the Fourier domain [6,7], significantly reducing the computational complexity compared to spatial-domain calculations.

The network is trained using image–pose pairs $\left\{\left(I,\xi^{\mathrm{gt}}\right)\right\}$ with a maximum likelihood objective. Given the end-to-end differentiable architecture, we minimize the negative log-likelihood loss:

(13) $$L=-\log P\left[\xi^{\mathrm{gt}}\right]$$

## 4. Experimental Results

### 4.1. Datasets

We conducted comprehensive experiments using two datasets: the Mapillary Geo-Localization (MGL) dataset [6] and the KITTI dataset [31].

The MGL dataset is a large-scale collection of geo-tagged images sourced from the Mapillary platform. It includes camera calibration data, noisy GPS measurements, and six degrees of freedom (6-DoF) poses in a global reference frame. Covering diverse urban environments under various conditions—different times of day, weather, and seasons—it comprises approximately 760,000 images from 12 cities. These images were captured using both handheld devices and vehicle-mounted cameras on cars and bicycles, providing a rich variety of perspectives and motion dynamics. For our experiments, we utilized a subset of around 360,000 images from five European cities (Montrouge, Toulouse, Le Mans, Nantes, and Vilnius) and one U.S. city (the Hayes area of San Francisco) for training. Additionally, 3987 images from Milan were reserved for testing in unseen regions to evaluate the method’s generalization capabilities. All images were resized and padded to a resolution of 384×512 pixels with a fixed focal length of 256 pixels, ensuring consistent input for our network and facilitating reliable feature extraction.

Serving as a widely-used benchmark, the KITTI dataset is particularly suited for evaluating performance in structured driving scenarios common in autonomous vehicle applications. It consists of driving sequences collected around Karlsruhe, Germany, encompassing urban areas, rural roads, and highways. Following the setup in [18], we employed this dataset for evaluation and comparison with state-of-the-art methods. Our training strategy involved pre-training on the MGL dataset, followed by fine-tuning on KITTI’s training split, which includes approximately 19,000 images. This approach leverages the diverse urban scenes in MGL for robust feature learning and adapts the model to KITTI’s specific driving scenarios. The test split comprises 7542 images from unseen regions, allowing us to assess the model’s performance in new and challenging settings. Images were resized and padded to 384×448 pixels with a focal length of 256 pixels to match the training input requirements.

We utilized map data from both Google Maps [11] and OpenStreetMap [9] to provide comprehensive environmental representations. Coordinates were initially provided in the WGS84 format (longitude and latitude). OSM data were rendered at a resolution of 0.5 m per pixel, offering detailed vector maps with 48 types of semantic information [6], including road networks and building footprints. The satellite imagery was constructed from map tiles obtained as 256×256 pixel images at zoom level 19.

### 4.2. Implementation Details

Our method was implemented using PyTorch and trained on an Intel i5-13600KF CPU paired with a single NVIDIA RTX 4070 Ti Super 16GB GPU. The model was trained with a batch size of 6 for 100,000 steps on the MGL dataset and fine-tuned on the KITTI dataset for 3000 steps using the AdamW optimizer with a fixed learning rate of $5\times 10^{-5}$. During training, we rendered and encoded a 192×192 map feature at a resolution of 0.5 m per pixel, with the ground truth (GT) pose centered within ±16 m from the map center, corresponding to a 64×64 pixel search region. To save GPU memory, we considered 96 BEV rotation angles over 360° during training.

We employed ConvNeXt-Tiny [32] as the street-view image backbone and a feature pyramid network (FPN) [33] to infer an 8× downsampled image feature. The transformer decoder comprised L=3 layers, h=4 heads, and a feature dimension of N=128. The hidden layer dimension in the feed-forward network was set to 512. The BEV covered a depth of 32 m with a resolution of 1 m per pixel (Δ=1), resulting in D=32 polar queries. The final BEV was upsampled to 0.5 m per pixel and matched with the map within c=8 channels. The details of the map encoding are provided in Figure 4.

### 4.3. Evaluation Metric

Our method estimates a 3-DoF camera pose by matching the BEV map against 2D maps. Following previous work [18], we evaluated the localization performance by reporting the camera’s position errors along the longitudinal (i.e., driving direction) and lateral directions separately, as well as the orientation error.

An estimated translation along a particular direction is considered correct if it is within *d* meters of its GT translation in that direction. Similarly, an estimated rotation angle is deemed correct if it is within θ° of its GT value. We set the distance thresholds *d* to 1 m, 3 m, and 5 m and the angular thresholds θ° to 1°, 3°, and 5°, respectively. The recall at threshold *X* (R@X) represents the percentage of correct estimations that fall within the specified threshold *X*.

### 4.4. Comparison to State of the Art

We compared our method against several state-of-the-art approaches:

- LM [18] is an iterative method for estimating the location and orientation of a ground camera on aerial images. It refines pose estimates by aligning features between ground and aerial views iteratively.
- SliceMatch [34] employs a slice-wise cross-view attention mechanism to generate *K* pose-dependent aerial image descriptors, which are then matched with a single ground image descriptor to determine the most accurate pose among the *K* candidate poses.
- CCVPE [35] generates orientation-aware descriptors to separately estimate both location and orientation in the aerial image, leveraging orientation-sensitive features to enhance pose estimation accuracy.
- OrienterNet [6] serves as our base framework, utilizing BEV-map matching to estimate the camera pose in OpenStreetMap using a CNN-based model.

As mentioned in Section 4.1, we report the performance of our method on the KITTI dataset using the Test2 split [18] for cross-area evaluation, with the number of rotations set to 512 during evaluation. Table 1 presents the recall rates at various thresholds for lateral and longitudinal positional errors, as well as orientation errors.

**Runtime Analysis:** On the same device (a single RTX 4070Ti Super GPU), our method localizes an image in the KITTI dataset in 88 ms (11 FPS) when using 512 BEV rotations. Specifically, BEV inference takes 5.7 ms (compared to 6.5 ms in OrienterNet), map encoding takes 3.7 ms (compared to 3.1 ms in OrienterNet), and matching takes 61 ms. This is slower than CCVPE’s 66 FPS but faster than LM’s 3.3 FPS. Theoretically, the matching calculation is high and grows linearly with the number of rotations, which is an inherent limitation of BEV-based matching methods. To address this, potential optimizations could focus on improving the efficiency of the matching procedure. For example, by leveraging an angle prior of ±10°, the total rotation range is reduced from 360° to 20°, thereby reducing the matching calculation to approximately 1/18 of the original.

**Positional Accuracy:** Our method achieves the highest recall rates across all thresholds for both lateral and longitudinal localization. Furthermore, pre-training on MGL significantly improved the localization performance [6]. Specifically, our method attains a lateral R@1 m of 77.96%, surpassing OrienterNet by 12.05%. Similarly, for longitudinal localization at 1 m, our method achieves a recall of 45.13%, significantly outperforming OrienterNet’s 33.07%. At broader thresholds of 3m and 5m, our method maintains superior performance with lateral recalls of 95.53% and 97.91%, respectively, compared to OrienterNet’s 92.76% and 96.54%.

**Orientation Accuracy:** In orientation estimation, our method achieves an orientation R@1° of 46.13%, outperforming OrienterNet’s 35.72%. At higher thresholds, our method continues to excel with R@3° of 87.40% and R@5° of 96.12%.

Notably, our method surpasses CCVPE in localization performance but has higher errors in orientation estimation. As mentioned in Section 4.2, during training, the orientation resolution was set to 360°/96 (approximately 3.75° per rotation increment for BEV), whereas during testing, the orientation resolution was increased to 360°/512 (approximately 0.70° per rotation increment). This discrepancy means that the model was not fully exposed to the finest orientation distinctions during training, potentially limiting its orientation accuracy.

### 4.5. Ablation Studies

To evaluate the effectiveness of both the proposed network and map fusion, we conducted two ablation studies to validate their contributions.

#### 4.5.1. Ablation Studies on Network Components

We conducted ablation studies to explore the impact of various components in our approach on localization performance using the MGL dataset in the Milan region. The results are summarized in Table 2. We defined four key components for these studies:

- T: Transformer-based BEV perception module. Without this component, depth distribution is applied to infer the BEV.
- W: Haar Wavelet Downsampling. Without this component, downsampling is performed using a 3×3 convolutional layer with a stride of 2.
- A: Spatial attention-based fusion strategy. Without this, feature maps are fused by simple addition.
- M: Multi-scale fusion strategy. Without this, fusion is defined as early fusion, where map features are fused together after the embedding layers for subsequent encoding.

The ablation studies demonstrate that each component contributes to enhancing localization and orientation performance. The full model, incorporating all components (T + W + A + M), achieves the highest recall rates across all metrics, with localization recalls of 16.96% at 1 m, 51.54% at 3 m, and 61.63% at 5 m, as well as orientation recalls of 29.82% at 1 degree, 65.76% at 3 degrees, and 77.10% at 5 degrees.

Removing the transformer module decreased the localization recall at 1 m from 16.96% to 16.30% and the orientation recall at 1 degree from 29.82% to 28.84%. This highlights the transformer’s crucial role in accurately inferring BEV features necessary for precise localization.

Excluding Haar Wavelet Downsampling resulted in a significant reduction in localization recall at 1 m to 15.65% and at 5 m to 58.11%, indicating that effective downsampling helps preserve critical feature information during map encoding.

Disabling the spatial attention-based fusion strategy led to a decline in localization recall at 1 m to 15.53%, suggesting that attention mechanisms enhance the fusion process by dynamically weighting feature contributions.

Removing the multi-scale fusion strategy caused the most significant performance drop, with localization recall at 1 m decreasing to 15.15%. This underscores the critical role of multi-scale fusion in capturing both global context and fine-grained details essential for robust localization.

Overall, the ablation results confirm that each component contributes to the model’s performance, with the Haar wavelet downsampling and multi-scale fusion strategies being particularly crucial for achieving high localization and orientation accuracy. The combination of these components enables the model to effectively integrate and leverage diverse feature information, leading to improved performance in cross-view geo-localization.

#### 4.5.2. Ablation Studies on Map Fusion

To further assess the effectiveness of our map fusion approach, we evaluated localization performance in the Milan region using different map inputs: only OpenStreetMap data, only satellite imagery, and both of them. The results are presented in Table 3.

**Positional Accuracy:** When trained exclusively on OpenStreetMap data, the model achieved a localization recall of 10.66% at 1 m, which increased to 13.59% with only satellite imagery. Satellite imagery alone provided better localization performance than OSM alone, likely due to the rich visual information aiding feature matching with ground-level views. Combining both OSM and satellite imagery for training, significantly improved performance, achieving a localization recall of 16.96% at 1 m—a 6.30% improvement over OSM alone and a 3.37% improvement over satellite imagery alone. Similar enhancements were observed at 3 m and 5 m, with recalls of 51.54% and 61.63%, respectively, compared to 37.20% and 48.16% with OSM alone and 45.75% and 56.73% with satellite imagery alone.

**Orientation Accuracy:** For orientation estimation, the model achieved an orientation recall of 23.85% at 1° using OSM data alone and 23.25% with only satellite imagery. The fused map improved this to 29.82%, representing gains of 5.97% over OSM alone and 6.57% over satellite imagery alone. At broader thresholds of 3° and 5°, the fused map achieved recalls of 65.76% and 77.10%, respectively, outperforming both individual map inputs.

These improvements indicate that combining OSM and satellite imagery not only enhances positional accuracy but also enables more precise orientation estimation. The subsequent Section 4.6 visualizes and further analyzes these enhancements.

### 4.6. Qualitative Results

**Figure 6:**
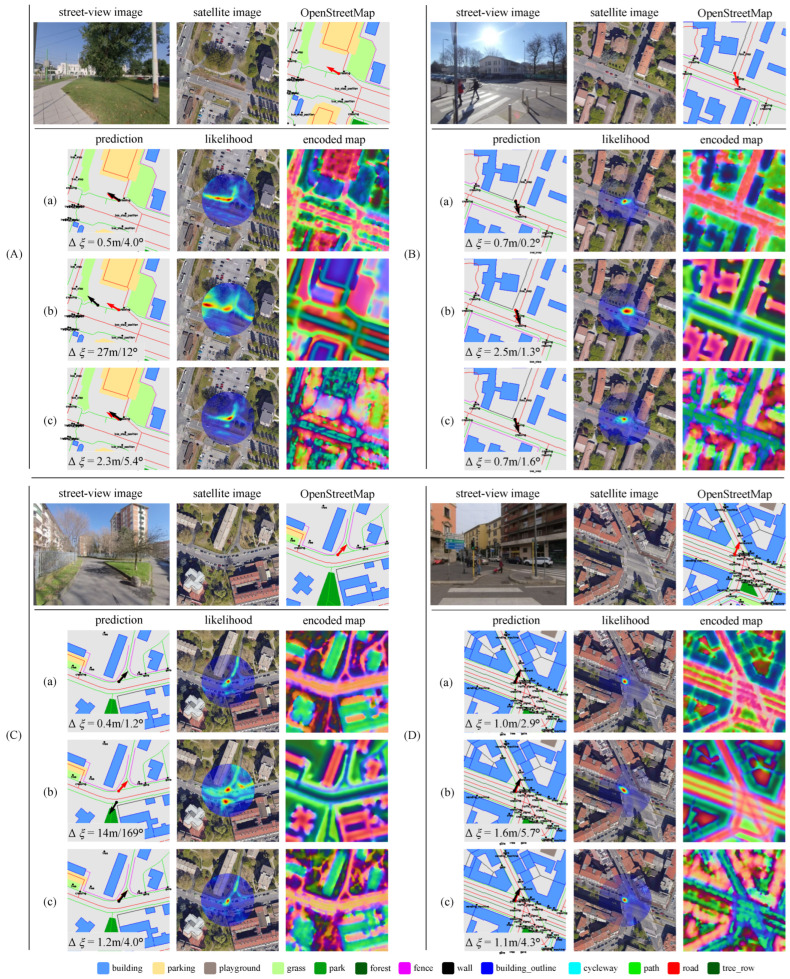
Qualitative results on the MGL dataset using three different map inputs on scenes (**A**–**D**) to visualize the shortcomings in OpenStreetMap. (**a**): Fused map for matching; (**b**): OpenStreetMap only; (**c**): Satellite imagery only. Red arrows denote the ground truth camera pose, while black arrows represent the predicted pose. The pose estimation error is denoted in the lower left corner of the prediction. Satellite maps data: Google, ©2024 Airbus, CNES/Airbus, Maxar Technologies [11].

**Figure 7:**
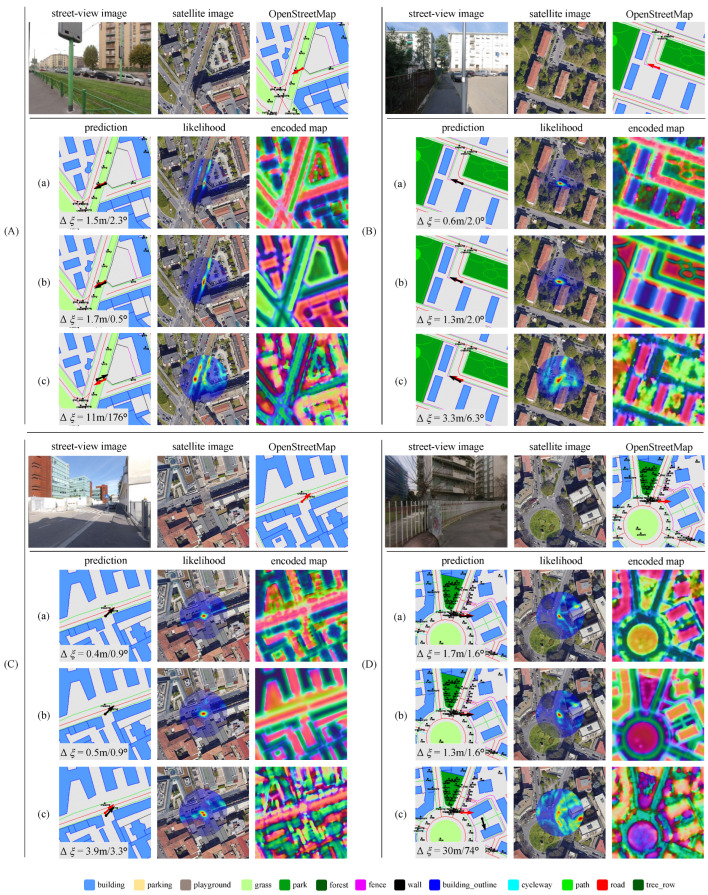
Qualitative results on the MGL dataset using three different map inputs on scenes (**A**–**D**) to visualize the shortcomings in satellite imagery. (**a**): Fused map for matching; (**b**): OpenStreetMap only; (**c**): Satellite imagery only. Red arrows denote the ground truth camera pose, while black arrows represent the predicted pose. The pose estimation error is denoted in the lower left corner of the prediction. Satellite maps data: Google, ©2024 Airbus, CNES/Airbus, Maxar Technologies [11].

To provide a comprehensive understanding of our method’s improvements resulting from map fusion, we present examples from the MGL dataset in the Milan region. Figure 6 and Figure 7 illustrate scenarios where BEV-map matching encounters challenges due to limitations in OSM data and satellite imagery, respectively. In these examples, the ground truth location is centered on the map within a 32-m radius search region. We visualize the encoded map by mapping the first three principal component analysis (PCA) values to RGB channels.

Figure 6 shows cases when using only OSM data. These examples primarily arise from incomplete or inaccurate OSM annotations, such as missing grass coverage (scenes A and C) and detailed road surfaces (scenes B and D). These omissions lead to multimodal pose likelihood distributions and large positional errors, as the model struggles to accurately match BEV feature with incomplete OSM data. In contrast, the incompleteness can be mitigated by fusing satellite imagery to enhance the robustness of localization.

Figure 7 illustrates cases that highlight the limitations of relying solely on satellite imagery. Specifically, the architectural diversity and complexity of high-rise buildings lead to ambiguous building coverage, as demonstrated in scenes A and D. Additionally, footpaths are often difficult to discern in satellite images but are clearly defined in OSM. This discrepancy can result in inaccurate pose predictions when satellite data are used exclusively. However, integrating OSM data addresses these challenges by providing explicit information on paths and buildings, significantly enhancing map feature construction, and supporting more robust localization.

These qualitative results emphasize the critical role of combining multiple map data modalities to improve localization performance. The fused map benefits from both OSM’s semantic richness and the visual detail of satellite imagery. In contrast, relying on a single data source can expose the model to specific limitations, such as incomplete annotations or visual inconsistencies. By merging these modalities, our method effectively mitigates the weaknesses of each individual source, ensuring more reliable and accurate pose estimations across diverse urban environments and varying capture conditions.

## 5. Discussion

**Figure 8:**
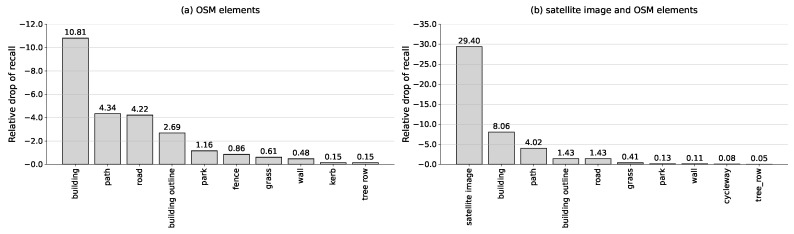
Relative drop of localization recall by removing different elements or satellite image from the map inputs.

In this section, we discuss the influence of various map elements from OpenStreetMap and satellite image on the matching process by examining their respective contributions to localization. Following the methodology of OrienterNet [6], we conducted ablation studies by systematically masking specific map elements from the OSM to assess the impact of each semantic class on localization accuracy. The results, depicted in Figure 8a, indicate that buildings, footpaths, and roads are the most valuable semantic classes for OSM-based localization.

Interestingly, as shown in Figure 8b, when satellite imagery is masked in map fusion-based localization, the importance of road diminishes significantly, while building and path continue to play a crucial role in accurate localization. This observation suggests that the detailed visual features captured in satellite imagery can compensate for the semantic information provided by certain map elements, such as roads. In contrast, masking the satellite imagery pixels by setting them to zero results in the largest relative drop in recall. This indicates that satellite imagery is the most critical factor for achieving accurate and reliable localization, outweighing other OSM elements in significance.

The fusion of OSM and satellite imagery leverages the strengths of both data sources. While OSM provides precise structural information about the environment, satellite imagery introduces rich visual textures, colors, and up-to-date details not captured in OSM data. This synergy enables the model to perform reliably under varying environmental conditions and ensures accurate localization even in areas where OSM data might be sparse or outdated. For instance, in regions with significant changes or less detailed OSM data, satellite imagery compensates by providing current visual information, thereby maintaining high localization accuracy. However, when scenes in satellite images are heavily obscured by clouds or fog, this enhancement becomes ineffective. This represents a common limitation of the CVGL method, as it inherently relies on the availability and quality of both spatial and visual data sources. Future work could explore integrating additional data modalities to mitigate the impact of such environmental factors.

## 6. Conclusions

In this paper, we introduced an enhanced version of OrienterNet [6] for cross-view geo-localization by fusing OpenStreetMap data with satellite imagery. This fusion leverages the explicit semantic information from OSM, such as buildings, alongside the rich visual details from satellite imagery, including roads, vegetation, and textures. By integrating these complementary data sources, we created a more informative and robust map representation tailored for navigation and positioning.

Our method effectively mitigates the limitations inherent in each individual modality. Semantic information from OSM aids in interpreting areas that are visually ambiguous in satellite imagery, while the rich visual details enhance the map’s discriminative capabilities. This combination enables more reliable feature matching between street images and the map, thereby improving localization accuracy. Consequently, this synergy leads to improved localization accuracy, as demonstrated by our experimental results, which show significant performance enhancements compared to using either data source alone.

Looking ahead, there is potential for further advancements. In future work, we plan to incorporate pseudo relative depth maps using advanced models [36] to generate a more robust BEV representation. Adding accurate depth information will provide additional geometric context, further enhancing BEV features and potentially increasing localization precision.

Our findings highlight the critical importance of integrating multiple remote sensing data modalities in cross-view geo-localization systems. By harnessing the strengths of diverse map sources, we pave the way for more resilient and accurate localization solutions under the urban scenes, fostering advancements in visual localization and autonomous navigation.

## Figures and Tables

**Table 1:** Cross-area evaluation on the KITTI dataset. The best performance is highlighted in bold. We report the recalls with lateral and longitudinal localization errors, as well as orientation errors, below specified thresholds. All methods are tested under a positional prior of ±20 m and an angle prior of ±10°. We also report results for OrienterNet and our method, both pre-trained on the MGL dataset. Baseline methods are sourced from their original papers. ✓: Pre-trained on MGL; ×: Not pre-trained on MGL.

Method	Pre-Trained on MGL	Lateral R@Xm			Longitudinal R@Xm			Orientation R@X°		
		**1 m**	**3 m**	**5 m**	**1 m**	**3 m**	**5 m**	**1°**	**3°**	**5°**
LM [18]	×	27.82	59.79	72.89	5.75	16.36	26.48	18.42	49.72	71.00
SliceMatch [34]	×	32.43	-	86.44	8.30	-	35.57	46.82	-	46.82
CCVPE [35]	×	44.06	81.72	90.23	23.08	52.85	64.31	**57.72**	**92.34**	**96.19**
OrienterNet [6]	×	51.26	84.77	91.81	22.39	46.79	57.81	20.41	52.24	73.53
Our Method	×	**68.48**	**90.82**	**94.94**	**31.84**	**61.44**	**69.61**	34.51	74.04	88.04
OrienterNet [6]	✓	65.91	92.76	96.54	33.07	65.18	75.15	35.72	77.49	91.51
Our Method	✓	**77.96**	**95.53**	**97.91**	**45.13**	**74.62**	**79.77**	46.13	87.40	96.12

**Table 2:** Ablation studies on BEV perception and map encoding. Positional priors are confined within a circular region with a radius of 32 m, and the queried map is centered on the ground truth to ensure a fair comparison. We report recall at various thresholds *X* (R@X) for localization errors (meters) and orientation errors (degrees). The highest performance in each category is highlighted in bold. ✓ indicates that the component is included, while × indicates that the component is excluded.

Component Choice				Localization R@Xm			Orientation R@X°		
**T**	**W**	**A**	**M**	**1 m**	**3 m**	**5 m**	**1°**	**3°**	**5°**
✓	✓	✓	✓	**16.96**	**51.54**	**61.63**	**29.82**	**65.76**	**77.10**
×	✓	✓	✓	16.30	50.29	61.12	28.84	65.29	77.03
✓	×	✓	✓	15.65	48.08	58.11	28.22	62.88	75.52
✓	✓	×	✓	15.53	49.79	59.64	27.94	63.18	74.97
✓	✓	✓	×	15.15	49.64	60.52	28.57	62.53	75.02

**Table 3:** Ablation study on map fusion. We report the recall at threshold *X* (R@X) for localization errors (meters) and orientation errors (degrees). The best results are highlighted in bold. ✓ indicates that the map input is included, while × indicates that the map input is excluded.

Map Data		Localization R@Xm			Orientation R@X°		
**OSM**	**Satellite**	**1 m**	**3 m**	**5 m**	**1°**	**3°**	**5°**
✓	×	10.66	37.20	48.16	23.85	55.46	67.49
×	✓	13.59	45.75	56.73	23.25	53.72	68.32
✓	✓	**16.96**	**51.54**	**61.63**	**29.82**	**65.76**	**77.10**

## Data Availability

Data are contained within the article.
